# MITOCHONDRIAL DNA COPY NUMBER IS ASSOCIATED WITH PHYSICAL FUNCTION

**DOI:** 10.1093/geroni/igad104.1965

**Published:** 2023-12-21

**Authors:** Qu Tian, David Zweibaum, Yong Qian, Richard Oppong, Jun Ding, Luigi Ferrucci

**Affiliations:** National Institute on Aging, National Institutes of Health, Baltimore, Maryland, United States; National Institute on Aging, National Institutes of Health, Baltimore, Maryland, United States; National Institute on Aging, National Institutes of Health, Baltimore, Maryland, United States; National Institute on Aging, National Institutes of Health, Baltimore, Maryland, United States; National Institute on Aging, National Institutes of Health, Baltimore, Maryland, United States; National Institute on Aging, National Institutes of Health, Baltimore, Maryland, United States

## Abstract

Mitochondria generate the energy required by cells to maintain functionality and muscle health. They may play a key role in age-related physical function decline. Skeletal muscle mitochondrial function is associated with gait speed and muscle strength. Blood-derived mitochondrial DNA copy number (mtDNAcn) is a biomarker of mitochondrial health, but its association with physical function is unclear. We examined cross-sectional associations of mtDNAcn estimated by whole genome sequencing with gait speed and grip strength in participants aged 50+ from two population-based studies: Baltimore Longitudinal Study of Aging(BLSA,n=688) and UK Biobank(n=150,448). Gait speed was measured using 6-meter walk in BLSA and self-reported walking pace in UK Biobank. Handgrip strength was measured using a hand dynamometer. We additionally tested sex differences because of known sex differences in gait speed and grip strength. We found that higher mtDNAcn was associated with faster gait speed and greater grip strength, after adjustment for age,sex,race,and autosomal coverage. Specifically, each standard deviation increase in mtDNAcn was associated with a 0.05 m/sec faster in gait speed and a 19% lower odds of reporting slow walking pace(both p< 0.0001). There was no significant sex-by-mtDNAcn interaction for gait (both p>0.05). There was a significant sex-by-mtDNAcn interaction for grip strength. In sex-stratified analysis, the association with grip strength was stronger in men (β=0.129,p=0.002;β=0.032,p< 2e-16,respectively) than in women (β=0.007,p=0.784;β=0.015,p< 2e-16,respectively). These findings suggest that blood-based estimate of mitochondrial function is related to physical function in community-dwelling middle-aged and older adults. Further research is needed to understand its relationship with physical function decline and underlying mechanisms.

